# The SHAP Explainer Model for Binary Classifiers Detecting Functional Groups in Molecules Based on FTIR Spectra

**DOI:** 10.3390/ijms27042004

**Published:** 2026-02-20

**Authors:** Tomasz Urbańczyk, Jakub Bożek, Jarosław Koperski, Marek Krośnicki

**Affiliations:** 1 Smoluchowski Institute of Physics, Faculty of Physics, Astronomy and Applied Computer Science, Jagiellonian University, Łojasiewicza 11, 30-348 Krakow, Poland; tomek.urbanczyk@uj.edu.pl (T.U.); jaroslaw.koperski@uj.edu.pl (J.K.); 2 Institute of Theoretical Physics and Astrophysics, Faculty of Mathematics, Physics and Informatics, University of Gdansk, Wita Stwosza 57, 80-308 Gdansk, Poland

**Keywords:** explainable AI, functional groups, FTIR spectra, neural networks, KAN

## Abstract

One of the disadvantages of deep learning models is the difficulty in understanding the premises on the basis of which the models make decisions. This might hinder the applicability of the model due to legal issues. In this paper, we investigate the decision process of CNN-KAN model trained to recognize chemical groups based on recorded FTIR spectra. The CNN-KAN model was trained as a binary classifier and using SHAP values, we could trace back the decision-making process and point out which part of the FTIR spectra are responsible for each positive or negative decision. It appears that the decision-making process of the deep learned CNN-KAN model is based on spectral regions that according to the literature have a large impact on the detection of particular functional groups.

## 1. Introduction

Nowadays, we are observing the rapid development of artificial intelligence (AI), especially models based on deep neural networks. Solutions based on AI are used to solve a wide range of problems in science and technology [1]. Some systems, especially those related to large language models, are used by humans on a daily basis [2]. However, deep learning models have certain limitations: due to their complexity and the number of training parameters, it is often impossible to understand why the model made the decision it did. Due to ethical and legal issues, this may limit the use of deep learning models in, e.g., finance [3] or healthcare [4]. In order to explain—in a human-understandable way—the premises on which deep models make their decisions, a new field of research has emerged: explainable artificial intelligence (XAI) [5]. XAI is commonly used in various fields (e.g., [6,7]), including molecular physics. The authors of Ref. [8] developed a machine learning model for the prediction of molecular dipole moments. This model indicates the premises behind the decisions made, e.g., by using attention maps. A common solution used in XAI involves employing a simpler explanation model, which serves as an interpretable approximation of the original complex model. One of the popular methods of creating explainable models is based on the game theory approach developed by Lloyd Shapley, i.e., the so-called Shapley values [9]. The idea behind Shapley values is to rightfully allocate credit for a model output among the input features of the model that is explained. In the case of binary classifiers, to each element of the input vector, a value can be assigned to determine its positive (positive value) or negative (negative value) influence on the model’s decision. Importantly, the absolute value of the assigned number determines the magnitude of the influence of a given element on the final decision of the model that is being explained. Recently, we have developed a classifier based on the CNN-KAN architecture [10], which was trained to recognize functional groups from Fourier-Transform Infrared (FTIR) spectra. To train the binary classifiers, we used one of the largest publicly available FTIR spectra databases in numerical form, which is provided by NIST [11]. FTIR is used on a regular basis for monitoring atmospheric gases based on characteristic vibrational fingerprints. On the other hand, designing new AI models and testing their decision-making process can influence the design of cheap field-based systems for environmental protection [12]. The aim of this work is to investigate the decision-making process of the CNN-KAN model used to predict the occurrence of functional groups based on the FTIR spectra.

## 2. Results

### 2.1. Interpretation of SHAP Results for Single Spectra

In this part of the article, several examples illustrate that the values returned by SHAP models correctly identify the regions of the spectrum that are characteristic of the functional groups under study.

**Figure 1:**
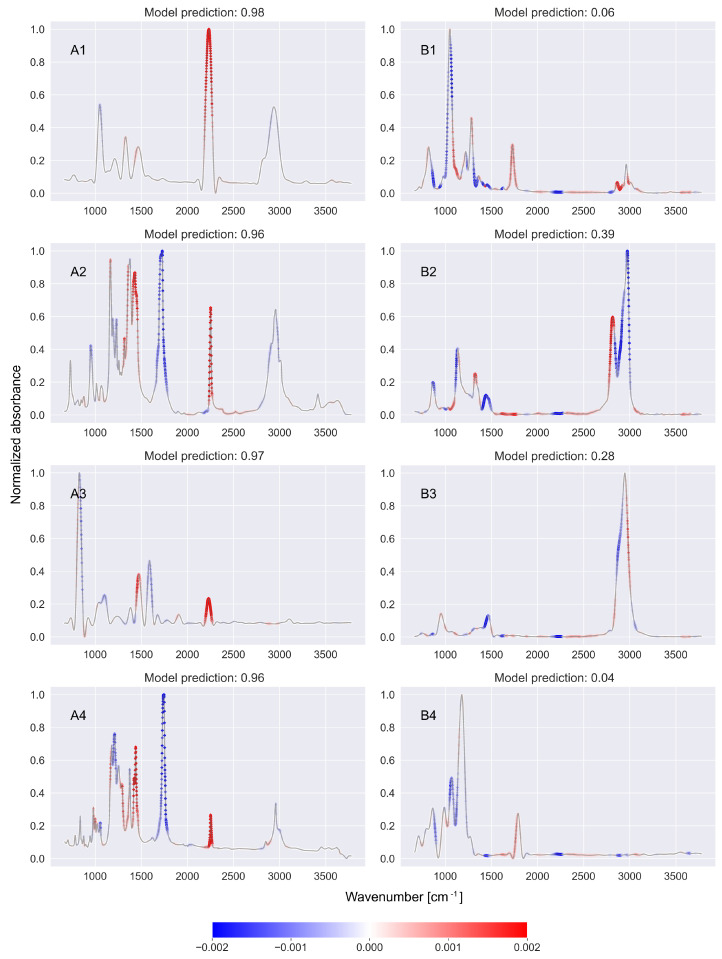
The values returned by the SHAP model based on the CNN-KAN classifier of the nitrile functional group in the FTIR spectra applied to the example spectra containing (**A1**–**A4**) and not containing (**B1**–**B4**) the nitrile functional group. The SHAP model properly indicated that the presence of a peak near 2200–2280 cm^−1^ (associated with C≡N bonding) is important for detecting a nitrile functional group. Further details can be found in the text.

**Figure 2:**
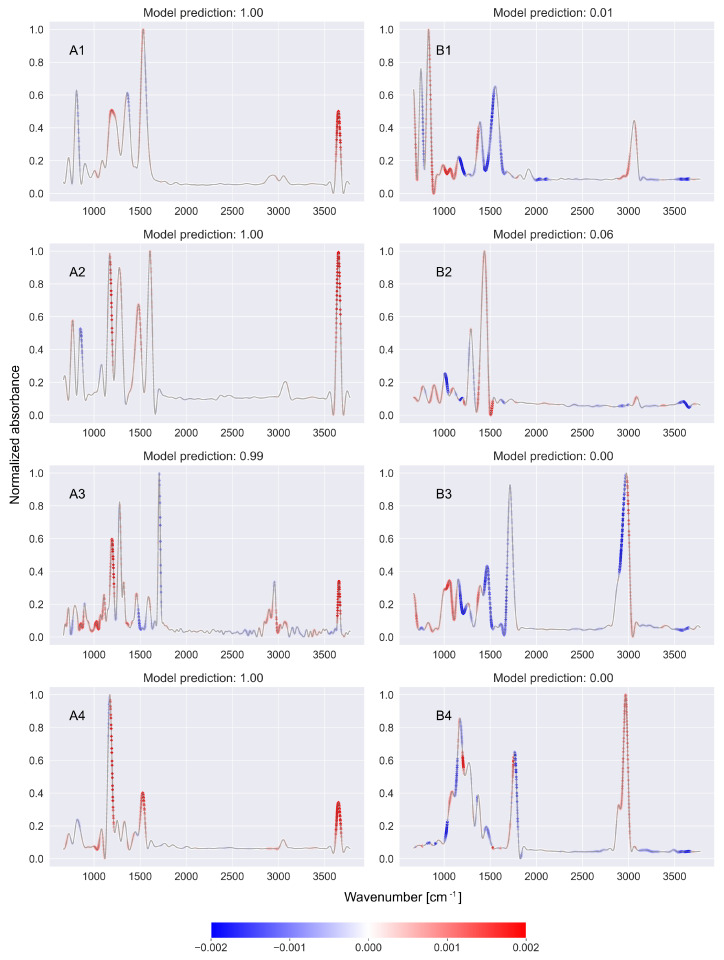
The values returned by the SHAP model based on the CNN-KAN classifier of the phenol functional group in the FTIR spectra applied to the example spectra containing (**A1**–**A4**) and not containing (**B1**–**B4**) the phenol functional group. The SHAP model properly indicated that the presence of a peak near 3200–3700 cm^−1^ (associated with O≡H bonding) is important for detecting a phenol functional group. Further details can be found in the text.

**Figure 3:**
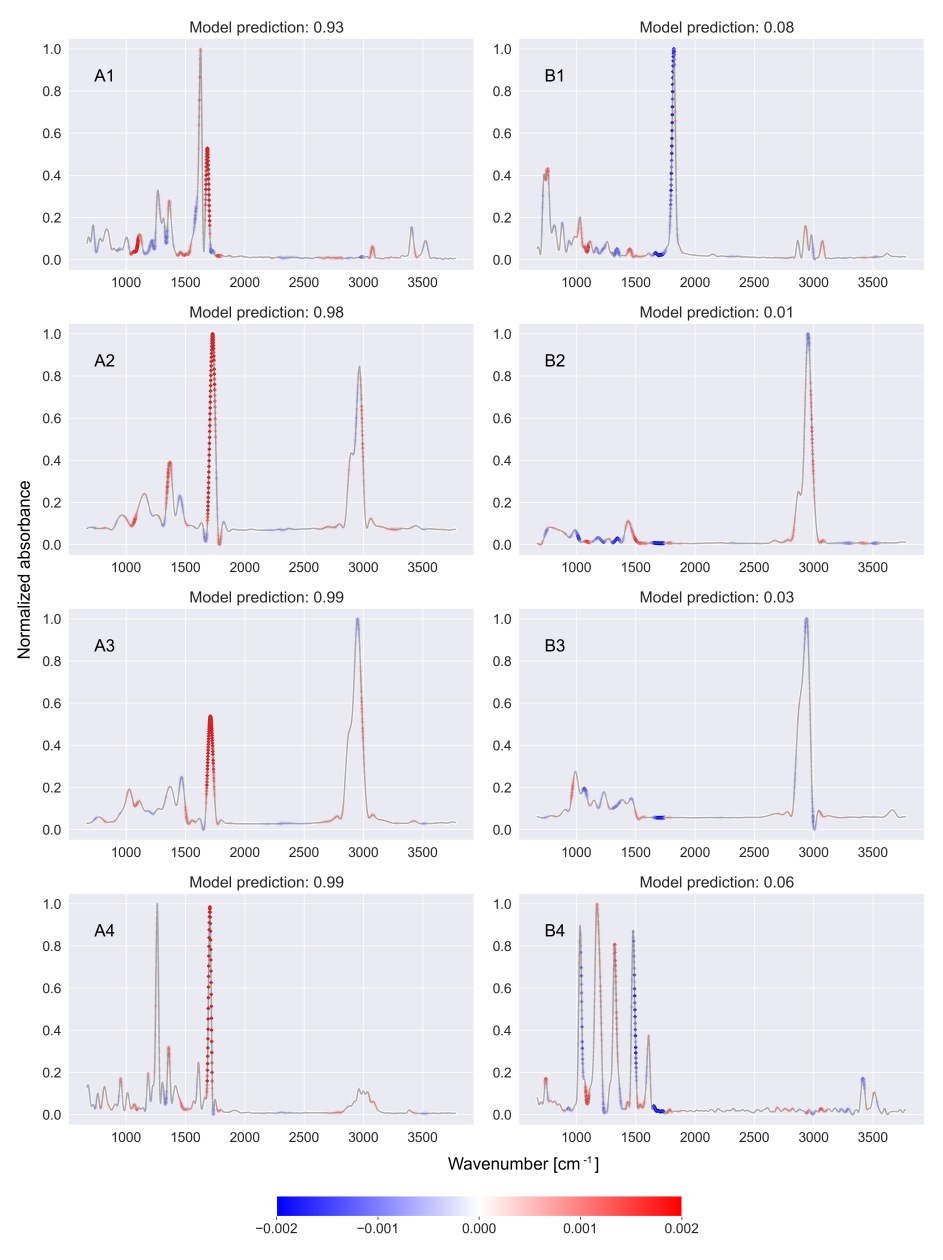
The values returned by the SHAP model based on the CNN-KAN classifier of the ketone functional group in the FTIR spectra applied to the example spectra containing (**A1**–**A4**) and not containing (**B1**–**B4**) the ketone functional group. The SHAP model properly indicated that the presence of a peak near 1650–1750 cm^−1^ (associated with C=O bonding) is important for detecting the ketone functional group. Further details can be found in the text.

Figure 1, Figure 2 and Figure 3 show the SHAP values returned by the SHAP model based on CNN-KAN classifiers detecting nitrile (Figure 1), phenol (Figure 2), and ketone (Figure 3) functional groups. In each figure, the left column (A1–A4) lists examples of spectra of molecules containing the analyzed functional group, while the right column (B1–B4) lists spectra of molecules that do not contain the analyzed functional group.

As mentioned earlier, the characteristic feature of the spectra of molecules containing the nitrile functional group is the appearance of a peak of energy in the range of 2200–2280 cm^−1^ [13]. According to Figure 1, the SHAP model correctly indicates the presence of a peak in the mentioned region as a premise that the spectrum originates from a molecule containing a nitrile functional group (large positive values returned by the SHAP model for the region 2200–2280 cm^−1^, which is visualized by the red points). Also, the absence of a peak in the mentioned range is interpreted correctly: it is a premise indicating that the spectrum comes from a molecule that does not contain a nitrile functional group (the SHAP model for the analyzed region of the spectrum returns large negative values, as visualized by the blue points).

Part A of Table 1 collects the summed values returned by the SHAP model for individual spectra from Figure 1 in the range of 2200–2280 cm^−1^. From this part of Table 1, it is clear that in the case of the presented spectra of molecules containing a group of nitriles, the sum of the values returned by the SHAP model in the specified spectral range is positive, while for the spectra of molecules not containing this group, the sum is negative.

In the case of phenols, the characteristic region of the spectrum is the range of 3200–3700 cm^−1^, where —OH stretching vibration bands occur [14]. As shown in Figure 2, the occurrence (spectra A1–A4) or absence (spectra B1–B4) of peaks in this area was correctly related by the SHAP model to the occurrence of the phenolic functional group in the molecule whose spectrum was being analyzed. The sums of values returned by the SHAP model in the range of 3200–3700 cm^−1^ for the spectra presented in Figure 2 are shown in Part B of Table 1.

For ketones, the characteristic region of the spectrum is the range of 1650–1750 cm^−1^, where there are peaks associated with the C=O stretch bond. Figure 3 shows that the explainable model correctly interprets this region as relevant for analyzing the occurrence of the ketone functional group. Compare this with Part C of Table 1, which shows the sums of the values returned by the SHAP model in the range of 1650–1750 cm^−1^ for the spectra presented in Figure 3.

### 2.2. Averaged Results for Exemplary Functional Groups

In the previous subsection, using the example of several spectra, it was shown that the SHAP models prepared for the CNN-KAN predictive models correctly indicate the fragments of the spectra that are relevant to check whether the given spectrum is associated with a molecule containing a functional group under study. This indicates that when making decisions, the CNN-KAN predictive models attach great importance to the same fragments of the spectra that would be considered in the case of classic manual analysis.

However, in order to graphically present the SHAP values returned for individual spectra in a convenient way, we have limited ourselves to only eight examples for each of the analyzed functional groups. To confirm our observation, in this subsection we perform an analysis on a much wider set of input data spanning 100 spectra for each functional group. It is worth noting that for each functional group, the test dataset was properly balanced—approximately half of the spectra in the test dataset were associated with molecules containing the functional group under study. In addition, for this subsection, we also expanded the number of analyzed functional groups from three to fourteen.

Table 2 presents two averaged values $S_{+}$ and $S_{-}$. $S_{+}$ is the averaged sum of the values returned by the SHAP model for the datapoints lying within the range (or ranges) characteristic for the analyzed functional group (see the second column of Table 2) and taking into account only those spectra from the test dataset that came from molecules containing the analyzed functional group. The algorithm used to calculate $S_{-}$ was similar; however, only those spectra from the test dataset that were associated with molecules that did not contain the analyzed functional group were taken into account. As one can see, for each functional group, $S_{+}$ takes positive values and $S_{-}$ takes negative values. From this we can conclude that for the analyzed functional groups, the regions of the spectra that—according to the literature—are relevant for the identification of a given functional group are indeed considered as important in the decision-making process of the CNN-KAN model.

At this point, it is worthwhile to explain why the absolute values of $S_{+}$ and $S_{-}$ for the nitrile, amide, and alkyne functional groups are clearly smaller than those for the other functional groups. This is most likely related to the fact that the prediction accuracy of the CNN-KAN model for these three functional groups was significantly lower than that for the other functional groups.

### 2.3. Identifying Regions Characteristic for Given Functional Group

In the previous parts of the article, we show that high positive or negative SHAP values occur in regions of the spectrum that are important from the point of view of determining whether or not the spectrum belongs to a molecule with a studied functional group. In this subsection, we show how to use SHAP values to determine so-called *fingerprint regions*—the regions of the spectrum that are relevant for studying the presence of a given functional group in a molecule.

For this purpose, the following procedure was used. For the functional group under study, the balanced test dataset containing 100 spectra was divided into two subsets: subset A contained spectra of molecules that had the functional group under study, while subset B contained spectra of molecules without a studied functional group. Then, for each subset, the SHAP values returned by the SHAP model for each of the spectra were summed and divided by the number of spectra. In other words, for each subset of the data, we calculated the average value of the SHAP parameters returned by the explainable model. Additionally, in the case of subset B, the result was multiplied by minus 1. In this way, two pointwise functions were obtained, namely, $F_{A}\left(x\right)$ and $F_{B}\left(x\right)$, which assigned a real value to each wavenumber *x* in the range of 650–3775 cm^−1^. It is worth noting that $F_{A}$ and $F_{B}$ functions are defined as averaged values of SHAP parameters for spectra belonging to the above-mentioned datasets. It also allows for easy calculation of standard deviations associated with individual points of the $F_{A}\left(x\right)$ and $F_{B}\left(x\right)$.

**Figure 4:**
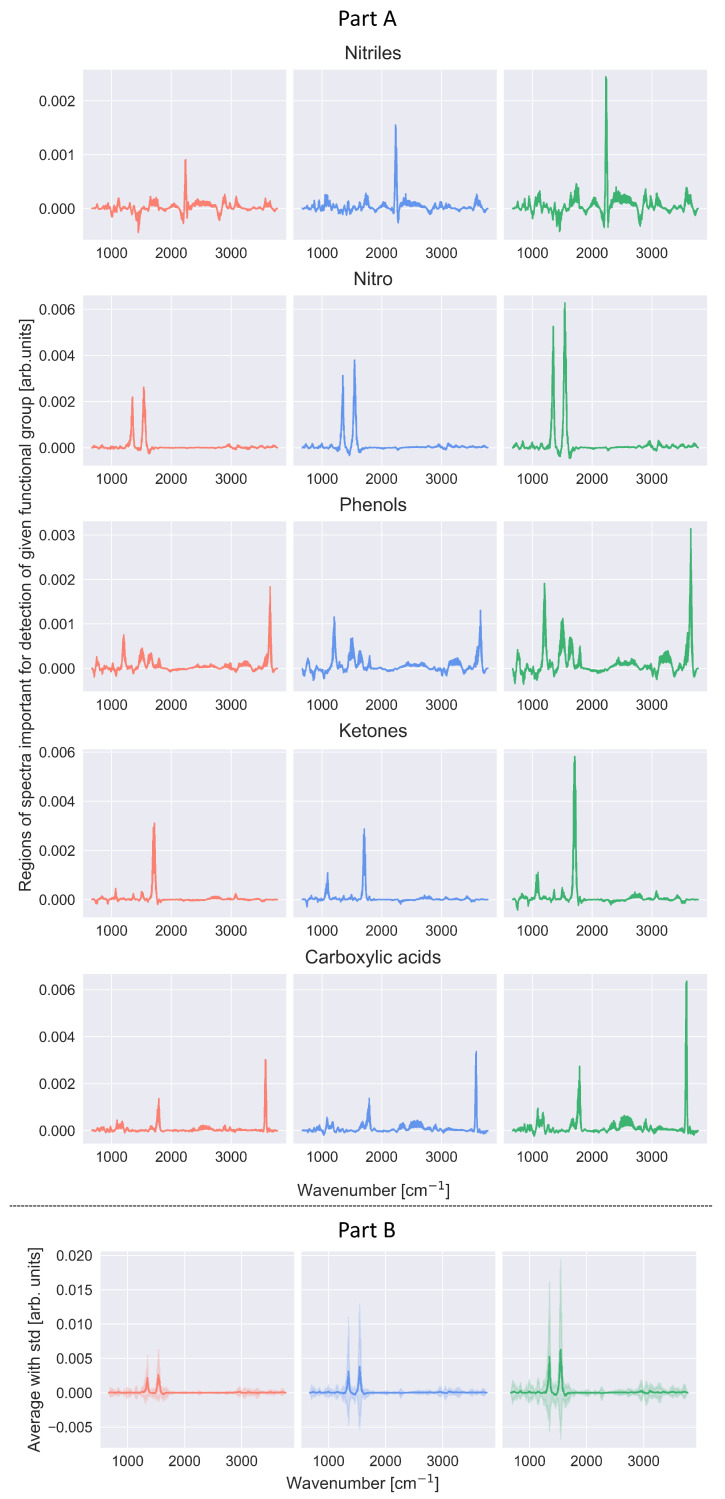
(**A**) illustrates characteristic spectral ranges for the functional groups nitriles, nitros, phenols, ketones, and carboxylic acids determined from the values returned by the SHAP explainable model applied to the CNN-KAN predictive model. (**B**) illustrates ± standard deviations (visible shades) of the $F_{A}$, $F_{B}$ and $F_{C}$ (solid lines) for the nitro functional group. Compare with the second row of (**A**). Further details can be found in the text.

The function $F_{A}$ takes high values in spectral regions that are important for determining the presence of a specific functional group in the spectra of molecules that actually contain the group under study. In contrast, the $F_{B}$ function takes high values in the regions relevant to determining the presence of the functional group in the spectra of molecules that do not contain this group. In general, the functions $F_{A}$ and $F_{B}$ are correlated and for a given functional group, both assume high values in the same regions of the spectrum, but there is no simple transformation from one function into another (compare Figure 4). Finally, the $F_{C}$ function was introduced, which is the sum of the functions $F_{A}$ and $F_{B}$: $F_{C}=F_{A}+F_{B}$. This function takes large values in the regions of spectra that are important for determining whether a given spectrum originates from a molecule containing the analyzed functional group.

Figure 4A shows the values of the functions $F_{A}$ (first column), $F_{B}$ (second column), and $F_{C}$ (third column) determined for the five example functional groups: nitriles, nitro, phenols, ketones, and carboxylic acids. Additionally, Figure 4B shows the results for the nitro functional group with standard deviation envelopes of the $F_{A}$, $F_{B}$, and $F_{C}$ functions.

The obtained results clearly indicate that the selection of important spectral regions by the SHAP model coincides with the occurrence of structures characteristic for individual functional groups (compare with Table 2). Thus, in Figure 4 for nitriles, a large peak in the range of 2200–2280 cm^−1^ is observed, corresponding to the aforementioned C≡N bond. For the nitro functional group, two strong peaks are visible: one has its center around 1350 cm^−1^ and the other at 1550 cm^−1^. They correspond to the N–O symmetric stretch in the region 1300–1390 cm^−1^ and the N–O asymmetric stretch in the region 1490–1570 cm^−1^. For the phenolic functional group, a wide maximum in the range of 3560–3670 cm^−1^ corresponding to the O-H bond is visible, as well as a peak with the center at about 1200 cm^−1^, which corresponds to the C–O bond. On the other hand, for ketones, a maximum in Figure 4 is visible for the energy at 1700 cm^−1^. It corresponds to the C=O bond.

### 2.4. Distinguishing Between Similar Functional Groups

In this part, it is examined whether the SHAP model can correctly distinguish between similar functional groups. Three functional groups were selected for the test: alkane, alkene, and alkyne. From a chemical point of view, alkane, alkene, and alkyne are hydrocarbons. Alkanes have at most single bonds (C–C) between carbon atoms. Alkenes have at least one double bond (C=C), while alkynes have at least one triple bond C≡C. The characteristic regions of the spectrum for these functional groups occur in the range of 2850–2990 cm^−1^ (alkanes), 3000–3100 cm^−1^ (alkenes), and 3200–3310 cm^−1^ (alkynes) [15].

**Figure 5:**
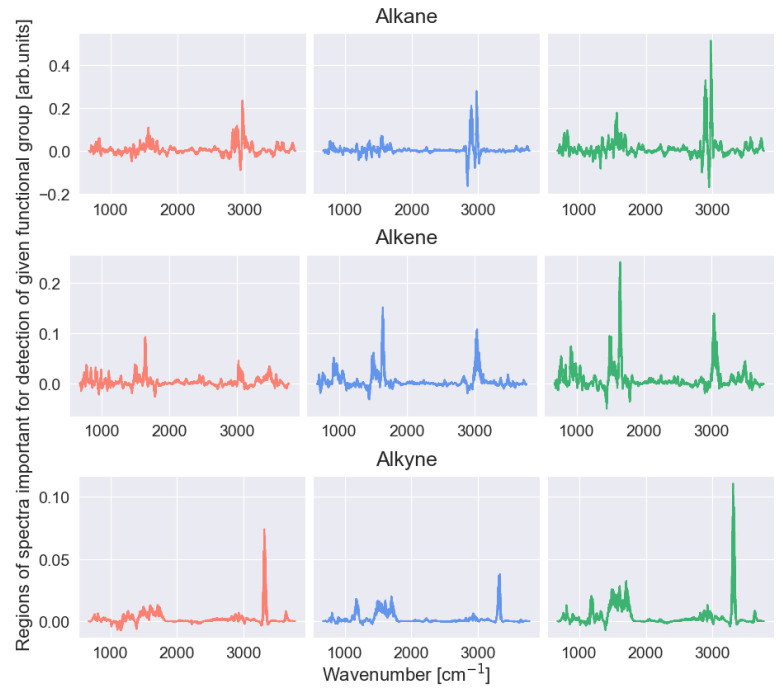
Characteristic spectral ranges determined from the values returned by the SHAP explainable model applied to the CNN-KAN predictive model for three similar functional groups: alkanes, alkenes and alkynes. Further details can be found in the text.

Figure 5 shows the regions of the spectrum that are important for the identification of alkanes, alkenes, and alkynes indicated by the explainable SHAP model. This figure shows the values of $F_{A}$, $F_{B}$, and $F_{C}$ in the same manner as in Figure 4 described in the previous subsection.

As one can see, the characteristic spectral ranges determined by the SHAP model for individual functional groups are clearly different (we focus here on the high-energy fragment of the spectrum lying in the region of 2850–3310 cm^−1^).

The characteristic range indicated for alkanes has the lowest energy and lies clearly below 3000 cm^−1^; the characteristic range for alkenes has a slightly higher energy, and the characteristic range for alkynes has the highest energy. This is consistent with the characteristic ranges given in the literature.

### 2.5. Results of the Explainable Model in Cases of Erroneous Decisions Made by the CNN-KAN Model

In real-world applications, a deep model classifier that is explained using an explainable model sometimes makes a misclassification. For example, for classifiers based on the CNN-KAN model, the prediction accuracy on the test dataset averaged across all functional groups is 84.4% (compare with Table 4 in Ref. [10]). The use of an explainable model can provide valuable insight into the premises on the basis of which the classifier made the wrong decision. This can help to understand the reasons why the deep model made a mistake.

**Figure 6:**
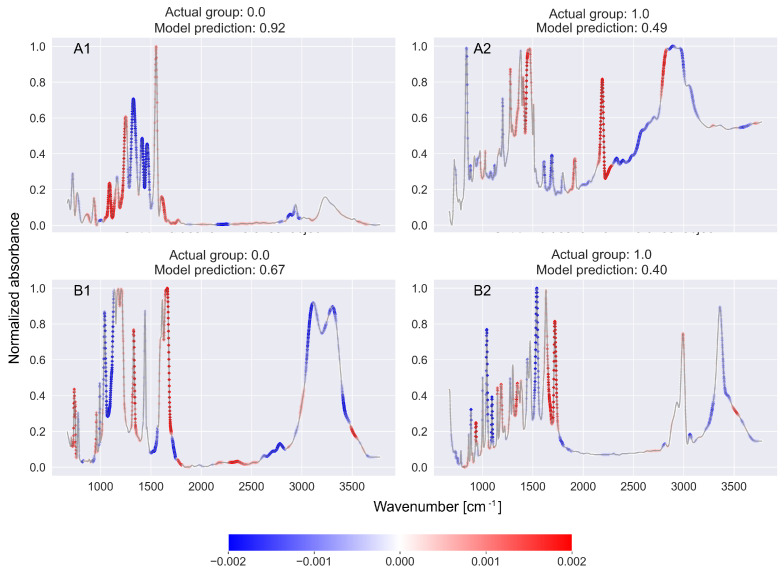
Examples of results of an explainable model for spectra for which the classifier made an erroneous decision. The spectra (**A1**,**A2**) refer to the detection of the nitrile functional group, while spectra (**B1**,**B2**) refer to the detection of the ketone functional group. In the cases of the (**A1**,**B1**) spectra, the CNN-KAN classifiers made false positive decisions, while for the (**A2**,**B2**) spectra, the decisions were falsely negative. Further details can be found in the text.

Figure 6 shows four spectra for which the CNN-KAN binary classifiers made incorrect predictions. The A1 and A2 spectra are associated with the detection of the nitriles functional group, while B1 and B2 spectra are associated with the detection of the ketone functional group. In the cases of the A1 and B1 spectra, the CNN-KAN classifiers made false positive predictions, while for A2 and B2 spectra the result was falsely negative. In the case of the A1 spectrum, the explainable model correctly indicates the absence of a peak in the nitrile characteristic region (2200–2280 cm^−1^) as a premise indicating that the CNN-KAN classifier’s decision should be negative. According to the explainable model, the premise for a positive decision of the CNN-KAN classifier is a peak around 1150 cm^−1^. This may indicate that the analyzed CNN-KAN classifier is undertrained, which may be related to the relatively small representation of spectra associated with the nitrile functional group in the training dataset (compare Tables 1 and 2 in Ref. [10]). For the A2 spectrum, the CNN-KAN classifier made a false negative decision, with the floating-point numerical value returned by the classifier equal to 0.49, which is very close to the threshold value of the classifier. In the case of this spectrum, the explainable model correctly indicated that the strong peak around 2200 cm^−1^ is a convincing indication for a positive decision of the CNN-KAN classifier. According to the indications of the explainable model, the ultimately negative decision of the CNN-KAN classifier was significantly influenced by a wide structure occurring above 2300 cm^−1^, which could confuse the classifier. In the case of the B1 spectrum, the falsely positive decision of the CNN-KAN classifier could have been influenced by the presence of a strong peak around 1650 cm^−1^, which the CNN-KAN classifier could associate with the C=O bonding characteristic for ketones. However, this bond is also found in carboxylic acids, amides, and aldehydes. Moreover, the C=C bond found in alkenes also has a similar energy. In the case of the B2 spectrum and a falsely negative decision of the CNN-KAN classifier, the interpretation of the indications of the explainable model is less clear. The presence of the peak in the vicinity of 1730 cm^−1^ associated with the C=O bond is in this case correctly interpreted as a prerequisite for a positive decision of the CNN-KAN classifier. The negative decision of the CNN-KAN classifier is supported by—according to the explainable model—narrow peaks in the regions of 1050, 1100, and 1550 cm^−1^, as well as a wide structure occurring above 3000 cm^−1^.

### 2.6. Tests of the SHAP Explainable Model on Other Dataset

In order to test the explainable models trained on the NIST dataset [11] on other dataset, we used the SWDRUG database [16] of substances used for drug production, which contains more than 800 ATR-FTIR spectra.

**Figure 7:**
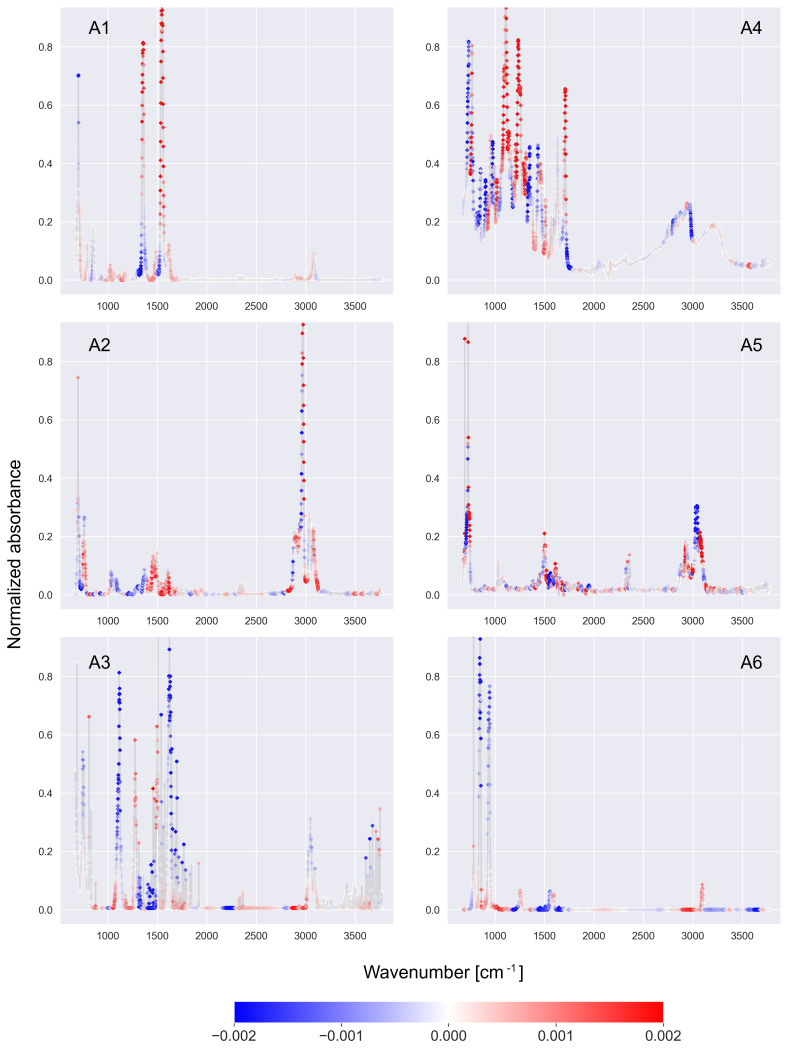
The SHAP values of explainable models calculated for spectra from the SWGDRUG Infrared Spectral Library [16]. (**A1**) Nitrobenzene spectrum tested on a nitro group classifier. (**A2**) Cumene spectrum tested on an alkane group classifier. (**A3**) Aniline spectrum tested on nitriles group classifier. (**A4**) Mitragynine spectrum tested on a ester group classifier. (**A5**) Toluene spectrum tested on an aromatic group classifier. (**A6**) Trichloroethylene spectrum tested on phenols group classifier. Further details can be found in the text.

The results of the tests are shown in Figure 7. For all the presented spectra, the CNN-KAN classifiers made the correct decision regarding the presence or absence of the studied functional groups. Part A1 shows the SHAP values calculated for the nitro group classifier for the nitrobenzene spectrum (this molecule contains the aforementioned functional group). The explainable model correctly indicated two strong peaks in the regions of 1350 cm^−1^ and 1550 cm^−1^ as a positive premise for the presence of the nitro functional group. Part A2 shows the SHAP values associated with the alkane classifier for the spectrum of cumene (this substance contains the functional group being analyzed). The explainable model correctly indicated a peak in the region of 2950 cm^−1^ (possibly associated with the C–H bond) as a positive premise for the presence of the alkane functional group in the cumene molecule. In the case of part A3, the aniline spectrum was tested on a nitrile functional group classifier, but this time the molecule does not contain the functional group under the study. The explainable model correctly interpreted the lack of the peak characteristic for nitriles associated with the C≡N bond (2200–2280 cm^−1^) as a premise that the studied molecule does not contain the nitrile functional group.

Part A4 shows the SHAP values for the mitragynine spectrum tested using the ester group classifier (where mitragynine contains the tested group). The explainable model correctly indicated the peak in the region of 1710 cm^−1^ (possible C=O bond) and peaks in the regions of 1100 cm^−1^ and 1240 cm^−1^ (possible C–O bond) as indications that mitragynine contains a group of esters. Part A5 shows the results for toluene when checking the presence of the aromatic functional group (toluen contains this group). The explainable model correctly interpreted the presence of peaks in the region of 700 cm^−1^ (possible C–H bond) and 1500 cm^−1^ (possible C=C bond), which lie in regions characteristic for the aromatic functional group. Part A6 shows the SHAP result obtained for the trichloroethylene spectrum tested on a phenols functional group classifier (the tested molecule does not contain this functional group). The explainable model correctly identified the absence of peaks in the region of 3200–3700 cm^−1^ as a premise that the molecule does not contain a phenolic functional group. The tests show that the SHAP explainable model has the ability to generalize and can also generate meaningful explanations for spectra from other datasets.

## 3. Materials and Methods

In this study, we used the SHAP framework [17] to explain the behavior of deep learning models (convolutional neural networks containing Kolmogorov–Arnold layers) [10], which were trained to predict the occurrence of 22 functional groups in molecules based on the FTIR spectra dataset. Importantly, the framework we chose has so far been used to create explainable models in practical applications such as medical data analysis [18], detection of faulty mechanical components [19], and even in the early prediction of cancer metastasis to lymph nodes [20]. The SHAP framework was also used academically in scientific research, e.g., to predict compound potency [21] and to interpret neural network pharmacokinetic models [22]. In the previously developed model [10] explained below, each neural network was trained separately and works as an independent binary classifier for the detection of a single functional group. The input vector of the model consists of 3106 elements containing the normalized FTIR absorption spectrum of the analyzed molecule in the range of 650–3775 cm^−1^. Each classifier returns one number, determining the probability that the analyzed molecule contains a specific functional group. Finally, the probability is rounded to 0-1 integers to obtain a binary result. The binary classifiers for each functional group have the same architecture. Details of the architecture of the classifiers can be found in Ref. [10].

It is worth noting that the machine learning models presented in Ref. [10] are an example of the so-called black-box models. Due to their complexity, it is virtually impossible to determine the premises on the basis of which the models determined whether a given spectrum is related to the molecule containing the studied functional groups or not.

In the classic approach (i.e., requiring the involvement of an experienced specialist), the classification of whether a given FTIR spectrum is related to a molecule containing a specific functional group is based on the analysis of the presence or absence of characteristic peaks in the spectrum. For example, the peak around 2280–2200 cm^−1^, associated with the C≡N bond, is a strong indication that a given spectrum comes from a molecule containing a group of nitriles, while its absence indicates that the studied molecule does not have this group [13]. It is worth noting that very often, the peak associated with a given chemical bond may appear in several functional groups, e.g., the C=O bond occurs, among others, in ketones, esters, and aldehydes, giving a peak in the range of 1680–1765 cm^−1^. However, it is sometimes possible to observe some small differences in the ranges for which the peak associated with a given bond is expected in the cases of individual functional groups [15]. Therefore, distinguishing between different groups may require an analysis of the simultaneous presence of several different peaks, or even the shape of a given peak (e.g., FWHM).

To explain the analyzed deep learning models, we used the GradientExplainer class from the SHAP framework. The GradientExplainer class uses the expected gradients method [23], which is an extension of the integrated gradients method [24], to explain model outcomes. This class allows for creating a model (hereinafter referred to as the SHAP model), that receives the same kind of data as the model that is being explained. The SHAP model assigns a real number (so-called SHAP value) to each element of the input vector. If the assigned number is positive, it means that a given element of the input vector contributes to the classifier’s positive decision; whereas if the assigned number is negative, this element favors the negative decision. The absolute value of the returned number describes how much influence a given element of the input vector has on the decision of the explained CNN-KAN model (the larger the absolute value, the greater the influence on the decision).

**Figure 8:**
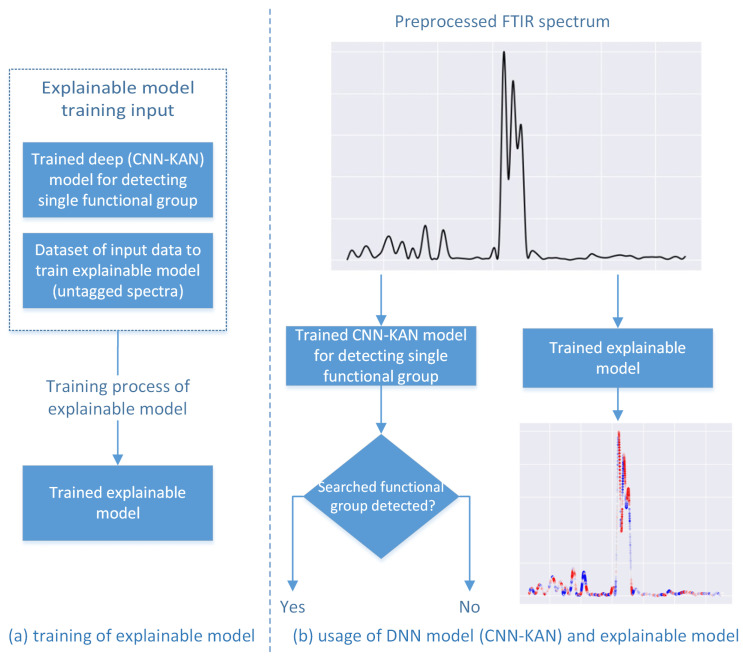
Diagram of using the SHAP explainable model. Further details can be found in the text.

The final result of the explainable SHAP model, which is easy for humans to interpret, is a spectrum with associated SHAP values. Each point of the spectrum can be plotted using a color map in which colors represent the values returned by the SHAP model for the given point (positive values correspond to red, negative values correspond to blue, while values close to 0 are represented in gray). Thanks to such a visualization, it is possible to determine which parts of the spectrum have an impact on the decision made by the deep learning model and to compare whether the premises that the explained model takes into account are similar to the premises that are taken into account in manual spectrum analyses. The results of such an analysis are described in the next section. Figure 8 shows a general scheme of the use of the SHAP model in our work, as well as the use of the deep learning model that is explained by the SHAP model. The training of the explainable models presented in this article was carried out using the aforementioned NIST FTIR spectra dataset [11].

## 4. Conclusions

Using the SHAP framework, we developed a model explaining the operation of the binary CNN-KAN classifiers [10] used to detect functional groups based on FTIR spectra. Using several functional groups as examples, it has been shown that spectral regions that according to the literature, have a large impact on the detection of particular functional groups, also significantly influence the decisions made by the CNN-KAN classifier. This means that the CNN-KAN model learned to make a decision based on physically important features of the spectra. Tests conducted on the drug precursor database [16] showed that explainable models trained on data from the NIST database [11] can work correctly on data from different sources, showing that the CNN-KAN model can generate inference beyond the datasets used for training and validation. The SHAP values analysis of the CNN-KAN decision process shows that classification is made on physically significant parts of the FTIR spectra. The model considers regions of FTIR spectra that are related to characteristical vibrations of the chemical groups.

However, a certain limitation of the explainable models shown here is the low prediction quality of the CNN-KAN models for some functional groups. It was often associated with the small number of spectra of molecules containing a given functional group that were available in the NIST dataset. The low prediction quality of the CNN-KAN models and the small number of spectra on which the explainable models were trained can negatively affect the quality of the results of the explainable models.

To the best of our knowledge, explainable AI models, including those using the SHAP framework, can be differently applied in the field of molecular physics. For example, they can be used to explain the reasons behind the decision made by deep learning models when determining physical parameters (e.g., dipole moments mentioned in the Introduction) on the basis of molecular data.

## Figures and Tables

**Table 1:** The sums of values returned by SHAP models trained for appropriate CNN-KAN classifiers for characteristic spectral regions for nitriles (2200–2280 cm^−1^), phenoles (3200–3700 cm^−1^) and ketones (1650–1750 cm^−1^) for spectra presented on Figure 1, Figure 2 and Figure 3, respectively.

Part A: Sum of SHAP values in characteristic region for nitriles 2200–2280 cm^−1^			
Spect. ID	Value	Spect. ID	Value
A1	0.36	B1	−0.46
A2	0.44	B2	−0.48
A3	0.45	B3	−0.39
A4	0.42	B4	−0.30
**Part B: Sum of SHAP values in characteristic region for phenols 3200–3700 cm^−1^**			
Spect. ID	Value	Spect. ID	Value
A1	0.13	B1	−0.32
A2	0.15	B2	−0.36
A3	0.14	B3	−0.19
A4	0.24	B4	−0.37
**Part C: Sum of SHAP values in characteristic region for ketones 1650–1750 cm^−1^**			
Spect. ID	Value	Spect. ID	Value
A1	0.19	B1	−0.36
A2	0.35	B2	−0.50
A3	0.24	B3	−0.32
A4	0.36	B4	−0.62

**Table 2:** The sums of the SHAP values calculated for characteristic ranges of spectra for fourteen functional groups averaged over test datasets containing 100 spectra. In the case of $S_{+}$, the averages were calculated for spectra associated with molecules containing the functional group under study, and in the case of $S_{-}$, the averages were calculated only for spectra associated with molecules not containing a given functional group. Further details can be found in the text.

Functional Group	Characteristic Range (s) ^a^ [cm^−1^]	$S_{+}$	$S_{-}$
Alcohols	1000–1300	0.16	−0.21
	3200–3650		
Aldehydes	1680–1715	0.21	−0.27
	2720–1740		
	2700–2900		
Alkane	1340–1395	0.12	−0.17
	1430–1480		
	2850–2990		
Alkene	685–995	0.12	−0.21
	1600–1680		
	3000–3100		
Alkyne	2100–2250	0.008	−0.009
	3200–3310		
Amides	1630–1700	0.07	−0.05
	3150–3500		
Amines	3250–3550	0.13	−0.08
Aromatics	680–900	0.44	−0.23
	1440–1620		
	3000–3100		
Carboxylic acids	1000–1300	0.13	−0.20
	1680–1725		
	3200–3500		
Esters	1000–1300	0.16	−0.27
	1715–1730		
	1735–1765		
Ketones	1650–1700	0.21	−0.20
	1705–1750		
Nitriles	2200–2280	0.03	−0.06
Nitro	1300–1390	0.35	−0.40
	1490–1570		
Phenols ^b^	3200–3700	0.14	−0.14

^a^ From Ref. [15], Table 20.2. ^b^ From Ref. [14].

## Data Availability

The data presented in this study are openly available in [GITHUB] at [https://github.com/marek-krosnicki/ftir-explainer].
